# Therapeutic impact of Nintedanib with paclitaxel and/or a PD-L1 antibody in preclinical models of orthotopic primary or metastatic triple negative breast cancer

**DOI:** 10.1186/s13046-018-0999-5

**Published:** 2019-01-11

**Authors:** Elaine Reguera-Nuñez, Ping Xu, Annabelle Chow, Shan Man, Frank Hilberg, Robert S. Kerbel

**Affiliations:** 1 Department of Medical Biophysics, University of Toronto, Toronto, Ontario Canada; 2 Biological Sciences Platform, Sunnybrook Research Institute, 2075 Bayview Ave, room S-217, Toronto, Ontario M4N 3M5 Canada; 3 Boehringer Ingelheim RCV, Vienna, Austria

**Keywords:** VEGF, Antiangiogenic drugs, Chemotherapy, Immune checkpoint therapy, PD-L1, Metastasis, Preclinical models

## Abstract

**Background:**

Triple negative breast cancer (TNBC) is an aggressive malignancy with poor prognosis, in part because of the current lack of any approved molecularly targeted therapy. We evaluated various combinations of three different drugs: nintedanib, an antiangiogenic TKI targeting VEGF receptors, paclitaxel (PTX), or a PD-L1 antibody, using models of orthotopic primary or advanced metastatic TNBC involving a metastatic variant of the MDA-MB-231 human cell line (called LM2–4) in SCID mice and two mouse lines (EMT-6 and a drug-resistant variant, EMT-6/CDDP) in immunocompetent mice. These drugs were selected based on the following: PTX is approved for TNBC; nintedanib combined with docetaxel has shown phase III clinical trial success, albeit in NSCLC; VEGF can act as local immunosuppressive factor; and PD-L1 antibody plus taxane therapy was recently reported to have encouraging phase III trial benefit in TNBC.

**Methods:**

Statistical analyses were performed with ANOVA followed by Tukey’s Multiple Comparison Test or with Kruskal-Wallis test followed by Dunn’s Multiple Comparison Test. Survival curves were analyzed using a Log-rank (Mantel Cox) test. Differences were considered statistically significant when *p* values were < 0.05.

**Results:**

Toxicity analyses showed that nintedanib is well tolerated when administered 5-days ON 2-days OFF; PTX toxicity differed in mice, varied with cell lines used and may have influenced median survival in the metastatic EMT6/CDDP model; while toxicity of PD-L1 therapy depended on the cell lines and treatment settings tested. In the LM2–4 system, combining nintedanib with PTX enhanced overall antitumor efficacy in both primary and metastatic treatment settings. In immunocompetent mice, combining nintedanib or PTX with the PD-L1 antibody improved overall antitumor efficacy. Using the advanced metastatic EMT-6/CDDP model, optimal efficacy results were obtained using the triple combination.

**Conclusions:**

These results suggest circumstances where nintedanib plus PTX may be potentially effective in treating TNBC, and nintedanib with PTX may improve PD-L1 therapy of metastatic TNBC.

**Electronic supplementary material:**

The online version of this article (10.1186/s13046-018-0999-5) contains supplementary material, which is available to authorized users.

## Background

Breast cancer, the most common malignancy in women worldwide, consists of four main subtypes: luminal A, luminal B, HER2-positive, and triple negative. This subgroup classification is based on expression of hormone receptors and overexpression of the human epidermal growth factor receptor 2 (HER2). Luminal A and B breast cancers are characterized by the expression of estrogen and progesterone receptors, with the main difference between them being the overexpression of HER2 in Luminal B cancers. On the other hand, HER2-positive breast cancers lack expression of hormone receptors, but overexpress HER2. Triple negative breast cancers (TNBC) neither express estrogen/progesterone receptors nor HER2, but it is now recognized to be a molecularly heterogeneous disease which can be classified in multiple subtypes, eg. basal-like 1, basal-like 2, luminal androgen receptor positive (LAR+) and mesenchymal-like [1–4]. Furthermore, the subtype of TNBC can have a significant impact on relative sensitivity to a particular drug or therapy such as cisplatin chemotherapy [1] and immunotherapy [4].

Breast cancer patients diagnosed with early stage Luminal A or B, or HER2-positive, have a better prognosis than TNBC patients, based in part on advances in development of hormonal and anti-HER2 therapies that target the estrogen/progesterone receptors and HER2, respectively. In contrast, there is not yet any targeted therapy approved to treat TNBC [5], in part related to its aggressiveness, high molecular heterogeneity, and non-specific inclusion in clinical trials, all of which can contribute to its poor prognosis [2, 6]. The only therapy currently approved for TNBC patients following surgery is cytotoxic chemotherapy, eg. using taxanes such as paclitaxel (PTX). Efforts continue in the quest to discover targeted therapies for TNBC [5–7].

Vascular endothelial growth factor (VEGF) is well known as a major inducer of angiogenesis [8]. Based on the contribution of angiogenesis for tumor development and progression [9], more than ten antiangiogenic drugs have been approved for over ten different types of cancer [10]. These drugs include VEGF pathway-targeting antibodies and antiangiogenic oral small molecule tyrosine kinase inhibitors (TKIs) targeting VEGF receptors (VEGFRs), among other receptor tyrosine kinases. There is evidence that inhibition of the VEGF pathway, for instance with bevacizumab (a VEGF monoclonal antibody) combined with chemotherapy, may have a benefit in metastatic breast cancer patients (mainly in the HER2-negative subgroup), based on results from the phase III clinical trials E2100 [11] and IMELDA [12]. Unlike VEGF pathway-targeting antibodies, most of the combinations tested in phase III trials involving antiangiogenic TKIs with various chemotherapeutic drug 'backbone' partners have failed to reach primary pre-specified endpoints in many different types of cancer, including breast cancer [13–26]. A recent exception is nintedanib combined with docetaxel as a second-line therapy for advanced non-small cell lung carcinoma (NSCLC) patients, as reported in a phase III trial called LUME lung-1 [27].

Nintedanib is a triple angiokinase inhibitor that targets VEGFRs, platelet-derived growth factor receptors and fibroblast growth factor receptors (FGFRs) -the three key pathways involved in angiogenesis-, and to a lesser extent, RET, Flt3 and Src [28]. This profile may contribute to its clinical efficacy and success. Another possible explanation is that it may be less toxic than other TKIs and thus combination with standard chemotherapy is more tolerable [29, 30], resulting in fewer instances of drug dose reductions or discontinuation of the combination treatment [13, 14, 17–19, 22–26, 31]. Indeed, nintedanib has been clinically evaluated in combination with different chemotherapy drugs in different types of cancer showing an acceptable safety profile [32–35].

Nintedanib has been evaluated combined with paclitaxel in a phase I clinical trial in early HER2-negative breast cancer patients showing an acceptable profile and promising antitumor efficacy [34]. Currently, there are two clinical trials testing nintedanib combined with paclitaxel (NCT01484080, phase I/II) and docetaxel (NCT01658462, phase II) in early and in metastatic or locally recurrent HER2-negative breast cancer, respectively.

In addition to its major role in angiogenesis, there is evidence that VEGF can act as an immunosuppressive factor by several mechanisms such as inhibiting dendritic cell (DC) function and maturation, enhancing expression of programmed death-ligand 1 (PD-L1) by DCs, promoting infiltration into the tumor of immunosuppressive T regulatory cells, tumor-associated macrophages and myeloid-derived suppressor cells, as well as inhibiting cytotoxic CD8+ T cell infiltration into tumors [36–39]. Thus, inhibition of the VEGF pathway may stimulate antitumor cell-mediated immunity, in addition to its effect on blunting angiogenesis. Combining drugs that target the VEGF pathway with immune modulators such as checkpoint inhibitors (eg. CTLA-4 and PD-1/PD-L1 antibodies) may enhance the antitumor effect of immunotherapy [40–43].

Immune checkpoint inhibitors such as PD-1/PD-L1 antibodies have been approved to treat multiple malignancies [44–52]; showing remarkable antitumor clinical effects, albeit only in minor subsets of patient (eg. 10–20%). Approximately 20% of TNBCs express PD-L1 [53], which has also been associated with tumor-infiltrating lymphocytes [54, 55] and response to neoadjuvant chemotherapy [55]. Furthermore, infiltration of T cells into TNBC tumors has been associated with a better outcome [54, 56–60]. The effect of PD-1/PD-L1 therapy has been evaluated in patients with advanced metastatic TNBC who had previously received chemotherapy and whose tumors express high levels of PD-L1 in two phase I clinical trials [61, 62], with values of objective response varying between 18.5% with pembrolizumab (a PD-1 antibody) [61] and 33% with MPDL3280A (a PD-L1 antibody) [62]. The effects of PD-1/PD-L1 therapy in metastatic TNBC could be enhanced when combined with chemotherapy, as recently reported for atezolizumab (a PD-L1 antibody) when combined with nab-paclitaxel in a randomized phase III trial (Impassion 130), although this regimen has not yet been approved [63].

Based in part on the immunosuppressive effects of VEGF, antiangiogenic drugs (which target the VEGF pathway) combined with immune checkpoint inhibitors are been tested in numerous phase II and III clinical trials; some studies show an increase in immune infiltrates along with promising antitumor effects in melanoma [64, 65] and renal cell carcinoma [43, 66, 67]. Indeed, there are currently a number of clinical trials evaluating antiangiogenic drugs (including nintedanib, NCT03377023) combined with immunotherapy in many different types of cancer [3, 68–70]. A supplementary table indicates the number and nature of ongoing phase II and III clinical trials of atezolizumab plus bevacizumab, mostly with other agents such as chemotherapy [see Additional file 1: Table S1]. However, despite reported clinical benefits, in some cases such combinations have resulted in substantial toxicity, especially with certain TKIs [71–73], presumably related to the target profile specificity of the antiangiogenic drug and the dose used, as well as previous exposure to different therapies.

The initial purpose of this preclinical study was to evaluate the hypothesis that combining nintedanib with paclitaxel would improve antitumor efficacy and survival in TNBC. The rationale for doing so was based on previous encouraging evidence using nintedanib to treat HER2-negative breast cancer patients [34] in addition to its apparent more tolerable safety profile compared to other TKIs such as sunitinib in other indications [29, 30]. To do so, we utilized an in vivo selected metastatic variant derived from the human TNBC cell line MDA-MB-231, called LM2–4. This way we could compare the results to those previously published using sunitinib (alone and combined with paclitaxel) to treat LM2–4 cells growing either as orthotopic primary tumors or as postsurgical advanced spontaneous metastatic disease [74]. These prior preclinical studies recapitulated failures of multiple phase III clinical trials evaluating sunitinib (alone and combined with chemotherapy) in metastatic breast cancer patients [18, 19, 26, 75]. We report that combining nintedanib with paclitaxel appears promising compared to prior sunitinib results, in this LM2–4 breast cancer model. Consequently, we next decided to evaluate this combination in additional models using syngeneic mouse breast tumors (namely, the EMT-6 cell line and a drug-resistant variant called EMT-6/CDDP) which would also allow us to test the two-drug combination combined with immune checkpoint therapy, in this case a PD-L1 antibody, in immunocompetent mice.

## Materials and methods

### Cell lines and mice

MDA-MB-231/LM2–4 is a variant of the triple negative human breast cancer cell line MDA-MB-231 (originally obtained from Dr. Jeff Lemontt, Genzyme Corp.) selected in vivo for its aggressive spontaneous metastatic properties after the established orthotopic primary tumor has been resected [76]. The LM2–4 cell line was cultured in RPMI 1640 medium with 5% fetal bovine serum (FBS) at 37 °C in 5% CO_2_, as previously described [76]. This cell line was authenticated to confirm its human origin by STR DNA analysis (Genetica DNA Laboratories). The EMT-6 (ATCC® CRL-2755™) mouse breast cancer cell line and the derived variant EMT-6/CDDP -selected in vivo for acquired resistance to cisplatin [77]-, were cultured in DMEM medium with 5% FBS at 37 °C in 5% CO_2._ All the cell lines were screened for mycoplasma contamination using commercial kits (Lonza) and were certified as being mycoplasma-free.

CB17 severe combined immunodeficient (SCID) mice expressing the yellow fluorescent protein (YFP CB17 SCID mice) were bred in house from breeding pairs originally provided by Dr. Janusz Rak (McGill University, Montreal). Balb/C mice were purchased from Jackson Laboratories. Mice were first used when they reached 6 to 8 weeks of age. All surgical procedures were undertaken in accordance with the animal care guidelines of Sunnybrook Health Sciences Centre (Canada) and the Canadian Council of Animal Care.

### Surgical procedures

Experiments performed with the MDA-MB-231/LM2–4 metastatic variant were done as described previously [76]. Briefly, 2 × 10^6^ cells of the MDA-MB-231/ LM2–4 cell line, were implanted in the mammary fat pad of female YFP CB17 SCID mice. To study the effect of drugs on tumor growth, treatment started once the primary tumor was established (average tumor size 150 mm^3^), around 14 days after cell implantation. Studies of metastatic disease treatment were undertaken after resection of primary tumors (average size 400 mm^3^) when the presence of overt metastasis is known (ie. beginning 3 weeks after tumor resection), based on previous studies with the LM2–4 metastatic variant [74, 76]. In this model, it is possible to observe metastatic nodules growing in the lungs, draining lymph nodes and/or liver during necropsy 3 weeks after tumor resection, with an incidence of 100% [74, 76]. All mice were randomized just before initiating treatment to obtain similar average tumor burden among groups.

For the mouse breast cancer models (EMT-6 and EMT-6/CDDP cell lines), 2 × 10^5^ cells were implanted orthotopically into the mammary fat pad of female Balb/C mice. To study the antitumor effect of drugs, the treatment was initiated when the primary tumor volumes reached 100-150 mm^3^, around 7 days after cell implantation. For studies of treatment of metastatic disease, primary tumors were resected when average tumor volume was 300 mm^3^, and therapy was initiated one week later, at a time when 100% of the mice have developed metastases in the lungs and draining lymph nodes, based on analysis of tissues during necropsy. All mice were randomized just before initiating treatment to obtain similar average tumor burden among groups.

Both for human and mouse breast cancer models, tumor growth was measured with Vernier calipers (once a week for LM2–4 and twice a week for EMT-6 and EMT-6/CDDP). Tumor volumes were calculated using the formula *a*^2^*b*/2, where *a* is the width and *b* is the length. Endpoint was considered when volume of primary tumors reached 1700 mm^3^. Mice were weighed daily to assess toxicity. In postsurgical treatment of advanced metastatic visceral disease, survival based on clinical symptoms was considered as endpoint.

To study possible mechanisms of action of the drug combinations, female Balb/C mice were used as recipients of orthotopically implanted 2 × 10^5^ EMT-6/CDDP cells. Treatment was initiated when the primary tumor volumes reached 100-150 mm^3^ (around 7 days after cell implantation) and all mice were sacrificed after 10 days of treatment (at a time when according to the primary tumor growth curve, some differences begin to emerge).

### Drugs and treatments

Nintedanib was provided by Boehringer Ingelheim (Vienna) and administered by gavage at the recommended dose 50 mg/kg, dissolved in double distilled water. Paclitaxel was purchased from Sunnybrook Pharmacy Department, Odette Cancer Center (Toronto, Ontario, Canada) at 6 mg/mL and further diluted with normal saline to the appropriate concentration, and administered intraperitoneally (ip) at 30 mg/kg 1q2weeks or at 50 mg/kg 1q3weeks, both considered as close to maximum-tolerated dose (MTD) [78]. PD-L1 antibody and its isotype were purchased from BioXCell (New Hampshire, USA) and administered ip at 5 mg/kg.

We analyzed the effect of nintedanib alone or when combined with paclitaxel and/or PD-L1 antibody, in both the primary tumor and the advanced metastatic treatment settings for breast cancer models, with the exception of LM2–4 xenograft models where PD-L1 antibody therapy was not undertaken. The treatment doses and schedules were as follows: 1) a control group treated with relevant vehicles and isotype control for anti-PD-L1 5 mg/kg ip 2q1week in experiments involving the PD-L1 antibody; 2) MTD PTX (50 mg/kg ip 1q3weeks in LM2.4 studies and 30 mg/kg ip 1q2weeks in EMT-6 and EMT-6/CDDP studies); 3) Nintedanib 50 mg/kg by gavage (po) daily (qd) for 2 weeks and then changed to 5-days ON, 2-days OFF; 4) the combination of PTX with nintedanib; 5) PD-L1 antibody (5 mg/kg) ip 2q1week; 6) the combination of nintedanib with PD-L1 antibody; 7) the combination of PTX with PD-L1 antibody; and 8) the triple combination: nintedanib, PTX and PD-L1 antibody. Due to toxicity observed in the advanced metastatic treatment setting with the LM2–4 cell line, after 2 weeks of treatment with nintedanib po qd, the schedule was changed to a 5-days ON, 2-days OFF. Mice receiving PD-L1 antibody showed signs of toxicity after the fourth dose, thus treatment was interrupted for one week and resumed for another 4 doses in mice implanted with EMT-6/CDDP cell line, or 1 dose in those implanted with EMT-6 cell line. Treatment groups varied depending on the experiment (eg. studies with the LM2–4 human breast cancer cell line grown in immunodeficient mice did not involve immunotherapy, as stated above).

### Histology and immunohistochemistry (IHC)

Tumors were fixed with 10% buffered formalin and embedded in paraffin. Tumor sections (5- μm-thick) were deparaffinized and stained with hematoxylin and eosin (Leica) to analyze necrosis. For IHC, sections were quenched in 1% H_2_O_2_ (except for CD8 staining where 0.3% H_2_O_2_ was used after secondary antibody), unmasked in boiling sodium citrate buffer (10 mmol/L, pH 6, 5 min), and stained using the following specific antibodies: CD31 (1:50, Dianova), Ki67 (1:400, Cell Signaling) and CD8 (1:100, Dianova). Biotin-conjugated secondary antibodies (Jackson ImmunoResearch) were used and detected with Vector Elite HRP kit and DAB chromogen (Dako). Sections were counterstained with hematoxylin (Leica). Sections were visualized with a Leica DM LB2 microscope and digital camera (DFC300FX) and images acquired using AxioVision 3.0 software. Images were analyzed using ImageJ 1.38d software.

### Proliferation assays

MDA-MB-231, LM2–4, EMT-6 and EMT-6/CDDP cells were plated in 96-wells culture plates (Thermo Fisher) (5000 cells/well for the human MDA-MB-231 and LM2–4 breast cancer cell lines, and 1000 cells/well for the mouse EMT-6 and EMT-6/CDDP breast cancer cell lines), in 100 μl of cell culture medium (RPMI 1640 for MDA-MB-231 and LM2–4 cells and DMEM for EMT-6 and /CDDP cells) containing 5% FBS. Cells were allowed to adhere overnight. Increasing concentrations of paclitaxel were added to the wells and the cells were incubated for 72 h at 37 °C in 5% CO_2_. Cell viability was checked using the MTS/PMS assay [3-(4,5-dimethylthiazol-2-yl)-5-(3-carboxymethoxyphenyl)-2-(4-sulfophenyl)-2H-tetrazolium, inner salt (MTS), in the presence of phenazine methosulfate (PMS)] from Promega. Tests were conducted in triplicates analyzing six wells per experiment.

### Statistical analyses

Statistical analyses were performed using the GraphPad Prism software package version 4.0 (GraphPad Software, Inc., San Diego, CA). Results are reported as means ± SD and were subjected to analysis of variance between groups (ANOVA). Following ANOVA, statistical differences between groups were evaluated by Tukey’s Multiple Comparison Test. In cases where data did not meet all the assumptions to use a parametric test, differences were evaluated with a Kruskal-Wallis test followed by Dunn’s Multiple Comparison Test. For immunohistochemistry and histology analyses, the data was subjected to non-parametric analysis using Mann-Whitney test. Survival curves were analyzed using a Log-rank (Mantel Cox) test. Differences were considered statistically significant when *p* values were < 0.05.

## Results

### Background to the overall rationale and use of the drug combinations tested

This preclinical study was initially designed to evaluate the combination of nintedanib and paclitaxel on TNBC using the LM2–4 human tumor xenograft system. This model was chosen in part since it would allow us to compare the results with those we previously obtained using sunitinib [74]. Based on the encouraging results obtained, especially when treating mice with advanced metastatic disease (as discussed below, Fig. 3a), we therefore decided to also evaluate this drug combination on mouse breast tumors using syngeneic models in immunocompetent mice. The rationale is that this would also allow us to include in the study an immune checkpoint inhibitor, a decision mainly based on clinical results that emerged during the course of our experiments with LM2–4 suggesting the potential benefit of targeting PD-1/PD-L1 pathway in TNBC [61]. Since only minor proportions of patient may benefit from the immune checkpoint therapy (eg. 20% or less); combination treatments could improve outcomes.

#### Nintedanib combined with paclitaxel delays growth of LM2–4 human breast cancer xenograft and improves median survival

Based on preliminary results of nintedanib combined with paclitaxel in a phase I trial treating early HER2-negative breast cancer patients [34], we decided to analyze the impact of nintedanib on advanced metastatic disease, both alone and combined with MTD paclitaxel in the LM2–4 model. Results using this metastatic model [74] recapitulated phase III failures involving advanced and metastatic breast cancer patients treated with sunitinib or sunitinib plus chemotherapy, including using paclitaxel, as discussed in the Introduction [18, 19, 26, 75].

**Fig. 1:**
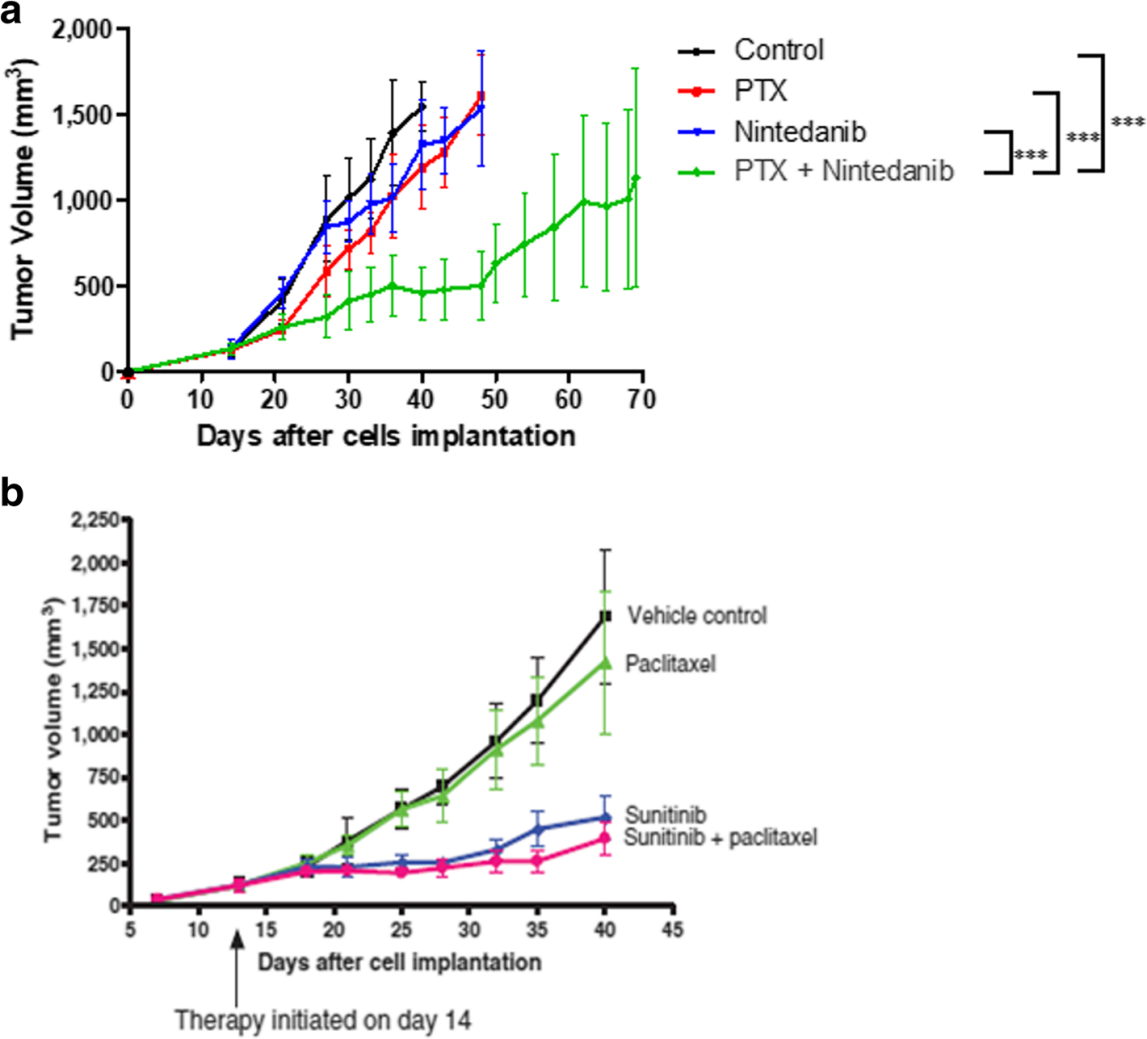
Nintedanib in combination with paclitaxel (PTX) delays tumor growth in LM2–4 primary tumors. **a**) Antitumor effect of nintedanib alone and when combined with PTX in the LM2–4 orthotopic primary tumor model. Nintedanib alone showed a very modest antitumor effect. Combination treatment resulted in the greatest inhibition of primary tumor growth. Treatments started after 14 days of cell implantation. Data are presented as means ± SD, *n* = 5. Statistical analysis on day 40 after cell implantation. ANOVA followed by Tukey’s Multiple Comparison Test, *** *p* < 0.001. **b**) Prior published results showing, in contrast, sunitinib having anti-tumor effect in this model, which was not improved by combination with PTX. Modified from Guerin et al.*,* 2013 [74]

**Fig. 2:**
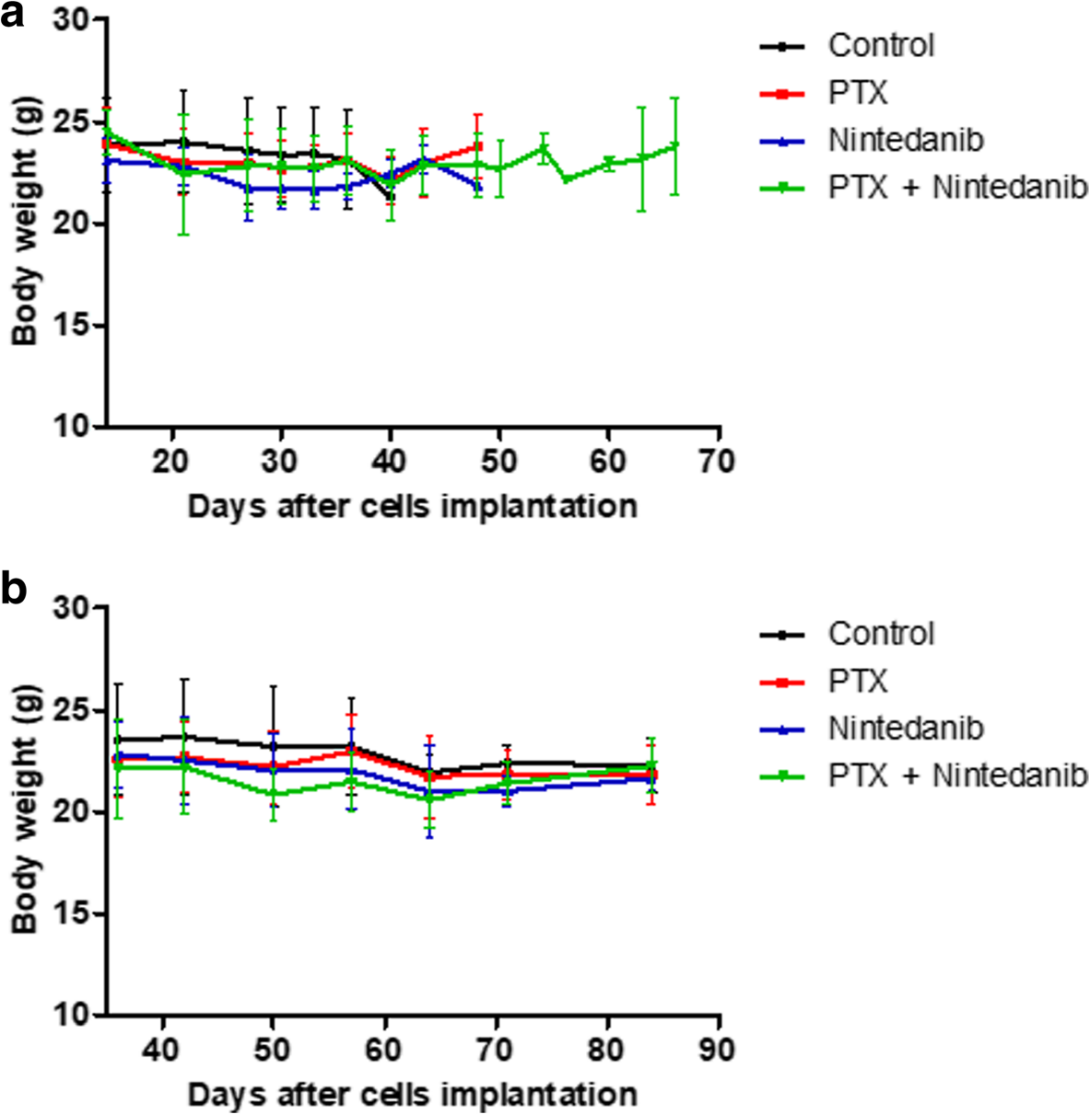
Toxicity of nintedanib, paclitaxel and the combination in the LM2–4 models. **a**) Primary tumor model. Different treatments were well tolerated. **b**) Advanced metastasis model. There were signs of toxicity in mice during the course of treatment (ie. scruffiness, hunched posture), but no significant weight loss. Body weight is considered as a surrogate for toxicity in mice. Data are presented as means ± SD, *n* = 5 (**a**) and *n* = 8–10 (**b**)

In this study, LM2–4 cells were implanted in the mammary fat pads of female SCID mice and treated with nintedanib, paclitaxel or the combination, both in primary tumor and advanced metastatic disease treatment settings. We observed that the combination treatment was the most effective in causing inhibition of primary tumor growth (Fig. 1a). However, somewhat surprisingly, nintedanib alone showed only a very modest delay of primary tumor growth, in contrast to the more potent antitumor effect previously reported using sunitinib or pazopanib in this same model [74] (Fig. 1b). We did not observe any signs of overt toxicity when mice were treated with nintedanib (Fig. 2a).

**Fig. 3:**
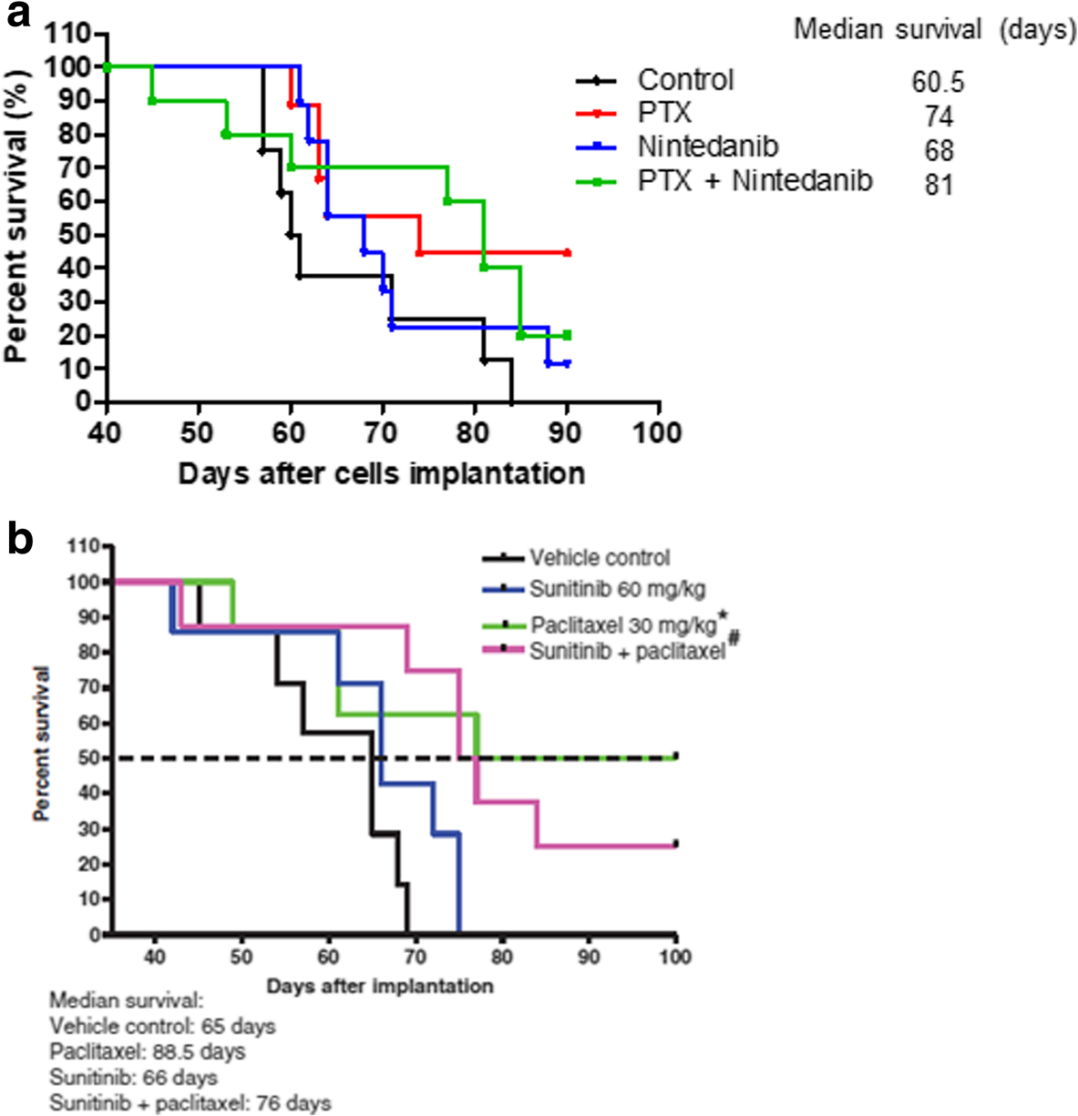
Paclitaxel and its combination with nintedanib increased median survival in the advanced metastatic breast cancer LM2–4 model. **a**) Kaplan-Meier survival curves and median survival values. Paclitaxel (PTX) significantly increased median survival compared to the control group (*p* = 0.033; Log-rank (Mantel Cox) Test, *n* = 8–10). Combination therapy increased median survival (81 days vs 60.5 days, control group) but it did not reach significance. Treatment started around 40 days after cell implantation. b) Effect of sunitinib alone and when combined with PTX in the advanced metastatic LM2–4 breast cancer model. Kaplan-Meier survival curves and median survival values. Modified from Guerin et al., 2013 [74]. PTX alone increased survival whereas sunitinib alone did not, and adding sunitinib to PTX did not result in increased efficacy

Nintedanib, paclitaxel and the combination were then evaluated on advanced metastatic disease after resection of LM2–4 primary tumors. The results indicated that the combination increased the median survival of mice compared to the control group (Fig. 3a). Although the increase in median survival did not reach statistical significance, this result is of considerable interest as it stands in contrast to what we observed previously in this same model using sunitinib and paclitaxel, which is recapitulated in Fig. 3b [74], where the combination treatment had a minor effect on survival. Two mice treated with nintedanib plus paclitaxel had to be sacrificed early in the experiment because of toxicity (as discussed below), which may have influenced the lack of statistical significance being reached in median survival despite marked improvement in this group compared to control (81 vs 60.5 days, respectively; Fig. 3a). The results suggest that nintedanib plus paclitaxel may have a beneficial effect when treating metastatic breast cancer compared to sunitinib plus paclitaxel, considering previous studies with this TKI (Fig. 3b), and the preliminary phase I clinical results discussed above [34].

#### Effect of nintedanib combined with paclitaxel and/or immunotherapy in primary tumor or metastatic mouse breast cancer models

Considering the aforementioned encouraging results of nintedanib combined with paclitaxel when treating LM2–4 advanced metastatic disease, we decided to evaluate this combination using the two mouse TNBC models. By using these syngeneic models, we were also able to evaluate nintedanib, paclitaxel and their combination with a PD-L1 antibody as a potential strategy to enhance overall antitumor efficacy of these drugs, considering also recent evidence suggesting the potential benefit of immunotherapy in TNBC patients [61–63].

For these studies, as discussed above, we used the EMT-6 mouse breast cancer cell line and a derived variant, EMT-6/CDDP, which was originally selected in vivo for acquired resistance to cisplatin by Teicher et al. [77]. The EMT-6/CDDP cell line does not show crossed resistance to paclitaxel, indeed it is significantly more sensitive to the drug than the EMT-6 cell line (IC_50_ 4.73 ± 1.32 ng/mL and 43.22 ± 6.08 ng/mL, respectively; *p* < 0.001), and similar to MDA-MB-231 and LM2–4 cell lines (IC_50_ 5.41 ± 1.83 ng/mL and 3.99 ± 0.78 ng/mL, respectively) [see Additional file 2: Figure S1]. We have found that EMT-6/CDDP cell line is more aggressive and metastatic, particularly to the lungs, compared to the parental cell line (unpublished observations). Also, we recently reported that this cell line expresses much higher levels of PD-L1 in vitro compared to the drug-sensitive parental cell line (EMT-6) [79].

**Fig. 4:**
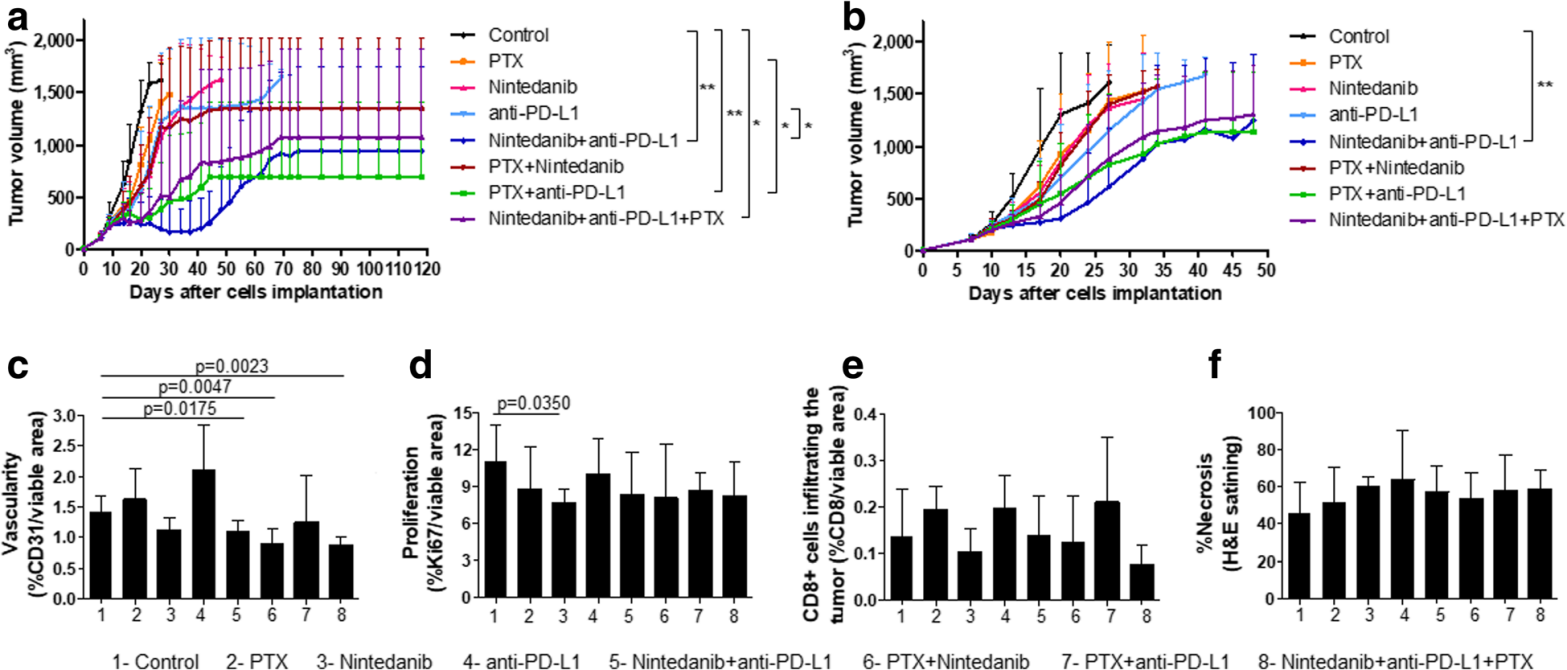
Improvement of immunotherapy efficacy when treating primary tumors with nintedanib combination therapy**. a**) Tumor growth in the primary EMT-6 breast cancer model. Treatment was started when average tumor volume was 120mm^3^, around 7 days after cell implantation. Statistical analysis on day 27. ANOVA followed by Tukey’s Multiple Comparison Test *p < 0.05, **p < 0.01. Data are presented as means ± SD, *n* = 6. Region of flat line in the curves means that tumor in remaining mice had regressed, and in the case of mice treated with PD-L1 antibody, tumors grew back. Mice were treated with nintedanib and/or paclitaxel (PTX) for 70 days, and then treatment stopped. b) Tumor growth in the primary EMT-6/CDDP model. Treatment was started when average tumor volume was 120mm^3^, 7 days after cell implantation. Statistical analysis on day 27. Kruskal-Wallis test followed by Dunn’s Multiple Comparison Test, **p < 0.01. Data are presented as means ± SD, *n* = 9–12. c-f) Effect of nintedanib, paclitaxel, anti-PD-L1 and the drug combinations on c) Vascularity; d) Proliferation; e) CD8+ tumor infiltrating cells; and f) Level of Necrosis. Histology and immunohistochemistry analyses were performed on tumor samples obtained from mice implanted with EMT-6/CDDP cells. Treatment was started when average tumor volume was 120mm^3^ and all mice were sacrificed after 10 days of treatment. The Mann-Whitney test was used for statistical analyses. Data are presented as means ± SD, n = 6–7

We first analyzed the effect of nintedanib, paclitaxel, a PD-L1 antibody and the various combinations on primary tumor growth. To do so, EMT-6 and EMT-6/CDDP cells were implanted in the mammary fat pads of female Balb/C mice. Mice were sacrificed when they reached endpoint because of tumor volume [see Additional file 3a: Figure S2a], at the time when most of them also have macrometastatic nodules in the lungs which are visible during necropsy [see Additional file 3b: Figure S2b] (although micrometastases can be found in all the mice, [see Additional file 3c: Figure S2c]). Surprisingly, in both cell lines, the combination of nintedanib with paclitaxel did not cause any benefit compared to either drug administered as monotherapy in the primary tumor treatment setting (Fig. 4a,b). These results stand in contrast with those obtained using the human breast cancer cell line MDA-MB-231/LM2–4 implanted in SCID mice (Fig. 1a), and with the sensitivity of the three cells lines to paclitaxel in vitro [see Additional file 2], as mentioned above, highlighting the contribution of the tumor microenvironment to the antitumor efficacy of the drugs.

All groups treated with PD-L1 antibody showed significant tumor growth delay in both mouse tumor cell lines (*p* < 0.05), compared to the control group (Fig. 4a,b), after the fourth dose (around day 20). In the primary tumor study using EMT-6 cell line, all the combinations involving PD-L1 antibody showed significant antitumor effect until day 27 (the time when all the mice in control group had reached endpoint). However, only the combination of PD-L1 antibody with nintedanib induced tumor regression that persisted until day 43 after cell implantation, when tumor growth resumed (Fig. 4a).

In the case of the more aggressive EMT-6/CDDP variant, the significant antitumor effect of PD-L1 antibody alone disappeared after one-week break off therapy. Only nintedanib combined with the PD-L1 antibody showed a significant antitumor effect compared to the control group until day 27 (the time when all the mice in control group had reached endpoint) (Fig. 4b).

In order to obtain insights into some possible mechanisms of action of the various drug combinations, a group of mice was implanted with the EMT-6/CDDP cell line, treated as in the primary tumor study and sacrificed after 10 days of treatment, at a time when some differences begin to emerge (Fig. 4b). Tumors in mice treated with nintedanib were less vascularized than tumors in the control group, reaching statistical significance when the TKI was administered in combination with paclitaxel and/or the PD-L1 antibody (Fig. 4c). In terms of proliferation (Fig. 4d), we observed a modest trend showing less proliferation, compared to the control group, in tumors treated with nintedanib or paclitaxel (as monotherapy or in combinations involving either of these drugs), the result being statistically significant only for tumors in mice treated with nintedanib alone. The infiltration of CD8+ cells into the tumors was very variable (Fig. 4e). There was no statistically significant change in the number of CD8+ infiltrating cells compared to the control group (Fig. 4e), although there was a trend showing increases in the number of these cells in tumors treated with paclitaxel or PD-L1 therapy. Such modest increases disappeared when nintedanib was added to the combination (ie. the triple combination) (Fig. 4e). In general, tumors were very necrotic (based on H&E staining) (Fig. 4f and Additional file 3a: Figure S2a), but with a trend to increase the level of necrosis for all the treatments evaluated (Fig. 4f).

**Fig. 5:**
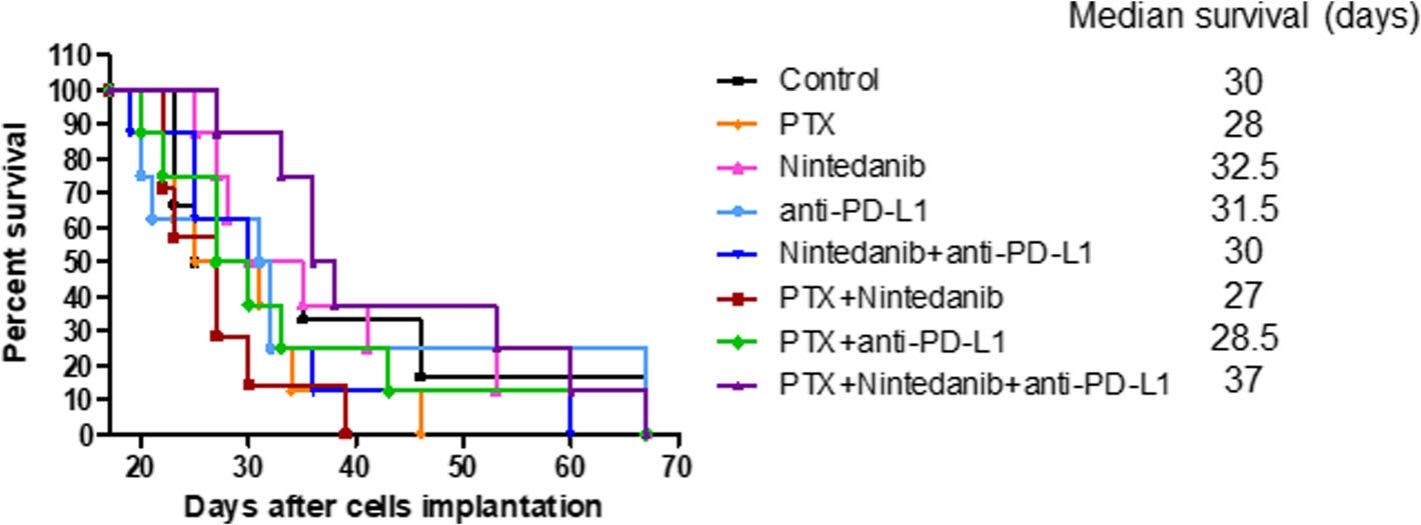
Nintedanib plus paclitaxel and PD-L1 antibody increased median survival in the advanced metastatic EMT-6/CDDP model. Kaplan-Meier survival curve median survival times in terms of days after cell implantation for each group. The triple combination improved median survival significantly compared to paclitaxel (PTX) alone (*p* = 0.0156) and nintedanib plus PTX therapy (*p* = 0.0125). All groups *n* = 8. Log-rank (Mantel Cox) test

Subsequently, to analyze the impact of nintedanib, paclitaxel, PD-L1 therapy and the combinations on advanced metastatic disease in immunocompetent mice, we followed similar procedures to that described for MDA-MB-231/LM2–4, ie. orthotopically implant EMT-6/CDDP cells, surgically resect the primary tumors, and then initiate treatment when overt visceral metastasis is present. Despite the prolonged median survival observed in the human LM2–4 xenograft system treated as advanced metastasis using nintedanib plus paclitaxel, we did not observe such a benefit in the mouse EMT-6/CDDP model (Fig. 5). Indeed, mice treated with this combination had a shorter median survival than the control group (27 vs 30 days, respectively), although it was not statistically significant.

We observed that nintedanib alone or when combined with PD-L1 antibody did not show any benefit when used to treat advanced metastatic disease in the EMT-6/CDDP cell line (Fig. 5), despite the antitumor effect induced by this combination in the primary tumor setting (Fig. 4b). Also, the tumor growth delay observed with PD-L1 therapy in the primary tumor model (either alone or combined with paclitaxel) (Fig. 4b) did not translate in an improved median survival in the metastatic treatment model (Fig. 5). In this case, the only relevant, significant improvement was observed when paclitaxel was added to the nintedanib plus anti-PD-L1 combination (ie. the triple therapy) (*p* = 0.0156), with respect to paclitaxel alone (Fig. 5). In general, mice treated with paclitaxel, alone or when combined with either nintedanib or the PD-L1 antibody, showed decreased survival outcomes compared to the control group, the exception being those mice treated with the triple combination. This discrepancy may be related in part to toxicity, which led to interruptions in treatment. These therapy breaks in some mice receiving paclitaxel (alone or combined with nintedanib or PD-L1 antibody) may have influenced the tumor burden; and this together with the general health status of mice likely contributed to the effects observed on survival.

#### Differential treatment toxicity profiles among cell lines and in primary tumor-bearing mice vs advanced metastatic disease settings

In the advanced metastatic treatment setting with the human TNBC cell line MDA-MB-231/LM2–4, we observed some signs of toxicity to nintedanib treatment when combined with paclitaxel (ie. mice general appearance, scruffiness, hunched posture), without significant loss of body weight (Fig. 2b). However, nintedanib was well tolerated in the primary tumor study, where LM2–4 cells were treated as tumors growing in the mammary fat pad of SCID mice. The reason for this is unknown. Therefore, after 2 weeks of daily dosing, the schedule was switched to 5 days per week (ie. 5 days ON, 2 days OFF), which resulted in significant improvement in mice general appearance, prolonged treatment and survival (Fig. 3a).

**Fig. 6:**
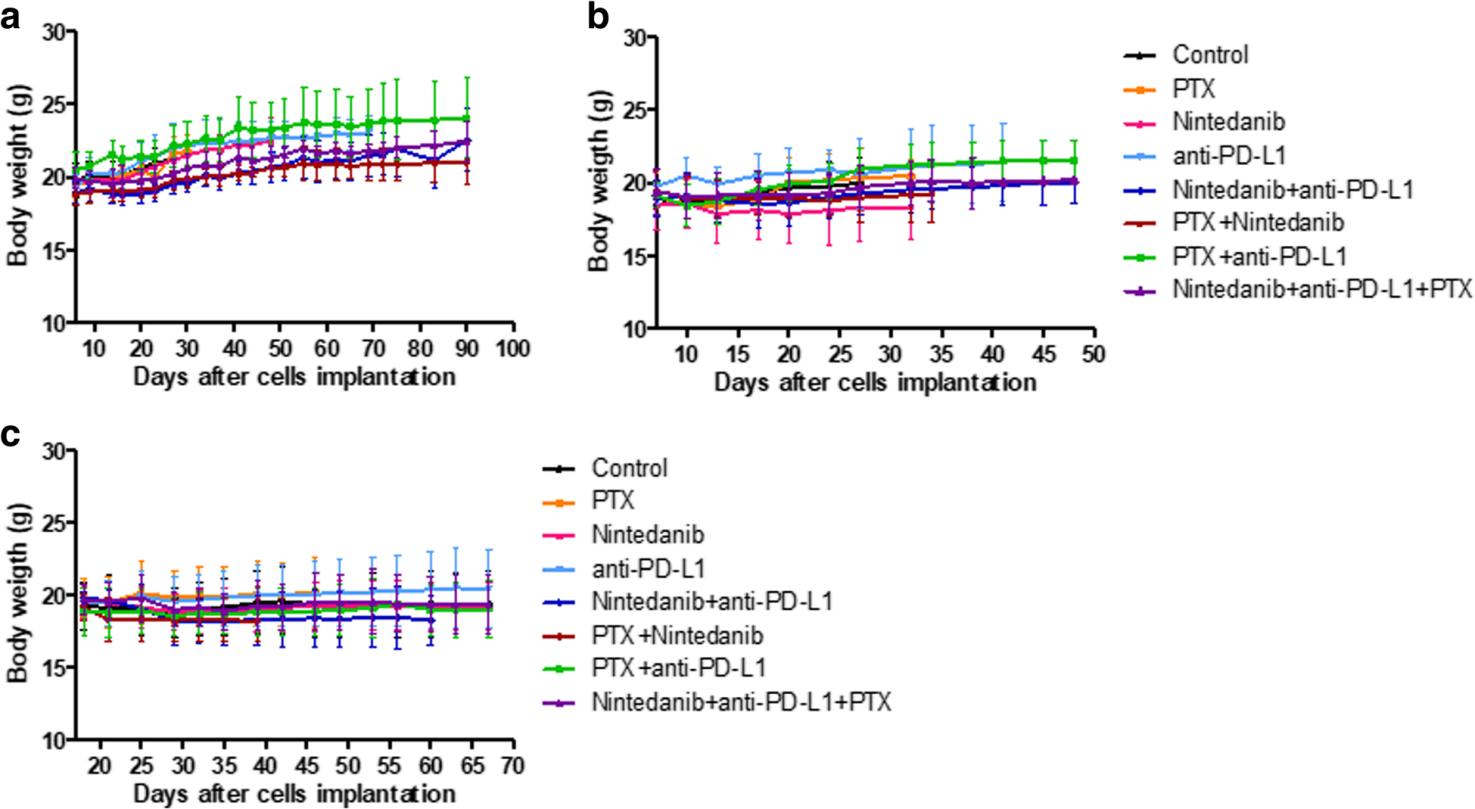
Toxicity of nintedanib, paclitaxel, PD-L1 antibody and the combinations in the EMT-6 and EMT-6/CDDP models. **a**) EMT-6 primary tumor model, **b**) EMT-6/CDDP primary tumor model, and **c**) advanced metastasis EMT-6/CDDP model. **a**) Treatments were in general well tolerated. **b** and **c**) Mice showed signs of toxicity in response to nintedanib and paclitaxel (PTX) at the beginning of therapy with no significant associated loss of body weight, but later they recovered. Toxicity associated with PD-L1 therapy did not affect body weight. Body weight is considered as a surrogate for toxicity in mice. Data are presented as means ± SD. *n* = 6 (**a**), *n* = 9–12 (**b**), *n* = 8 (**c**)

Mice implanted with the EMT-6/CDDP variant showed signs of toxicity to nintedanib and paclitaxel at the beginning of therapy with no significant loss of body weight associated, but later they recovered (Fig. 6b, c). This was not observed for the EMT-6 cell line in Balb/C mice (Fig. 6a) nor for the human breast cancer cell line (MDA-MB-231/LM2–4) growing as primary tumors in SCID mice (Fig. 2a), as mentioned above.

Regarding treatment with the PD-L1 antibody, we observed different toxicity profiles between EMT-6 and EMT-6/CDDP cell lines growing in mammary fat pads of female Balb/C mice. Mice showed signs of toxicity after four doses of PD-L1 antibodies, but only a few mice did not recover. Mice with EMT-6/CDDP primary tumors tolerated well PD-L1 rechallenge after a one-week break; whereas mice with EMT-6 primary tumors showed some toxicity after the rechallenge and received only 5 doses of PD-L1 antibody in total. Despite mice with EMT-6 primary tumors showing more toxicity in response to PD-L1 treatment than EMT-6/CDDP tumor-bearing mice, the antitumor effects induced were better after 5 doses of PD-L1 antibody when combined with nintedanib or paclitaxel. Toxicity events were more frequent in primary tumor-bearing mice. Mice with EMT-6/CDDP advanced metastatic disease did not show signs of toxicity after PD-L1 treatment. The basis for this difference is unknown, although it may be related in part to tumor burden and inflammatory response. Regardless, the results indicate that therapy studies in mice involving treatment of primary tumors versus metastatic disease on the other hand may yield very different outcomes- similar to the differences in efficacy outcomes.

## Discussion

For reasons outlined in the Introduction, the main purpose of this study was to evaluate the effects of nintedanib, paclitaxel chemotherapy, an immune checkpoint therapy (eg. a PD-L1 antibody) and their various combinations for efficacy and toxicity in several models of TNBC. Perhaps the most important finding of this study is the potential value of the triple drug combination (using nintedanib, paclitaxel and a PD-L1 antibody) in treating overt metastatic TNBC. Our results also highlight the potential of combining anti-PD-L1 therapy with nintedanib or paclitaxel to improve the overall antitumor efficacy of these drugs in TNBC, as well as the importance of utilizing preclinical models that involve treatment of not only primary tumors but also advanced metastatic disease.

The decision of evaluating nintedanib combined with immunotherapy was, in part, based on encouraging data suggesting that this TKI does not induce a significant myelosuppression nor affect the tumor infiltration of CD8+ T cells, in contrast to sunitinib (our unpublished observations, Fig. 4e). Of both interest and importance, our preclinical results, in general, are in line with recent clinical data from phase III trials involving both the use of a doublet treatment combining PD-L1 therapy and Nab paclitaxel to treat metastatic TNBC [63], and of a triplet therapy involving antiangiogenic, chemo- and immunotherapeutic (PD-L1 antibody) drugs, albeit for metastatic NSCLC [80]. This suggests potential predictive value of our models for the use of nintedanib in TNBC.

We initiated our studies evaluating the effect of nintedanib alone or combined with paclitaxel using the metastatic variant called LM2–4, derived from the human TNBC cell line MDA-MB-231 [76]. The very modest effect of nintedanib on tumor growth delay we observed (Fig. 1a) stands in contrast to the more potent antitumor effect previously observed by us [74] and others [81], when primary breast tumors xenografts, including LM2–4, were treated with other antiangiogenic TKIs (eg. sunitinib (Fig. 1b). Such differences may be related to the differential target profile specificities of sunitinib and nintedanib. Sunitinib targets a broader spectrum of receptor tyrosine kinases [82], compared to nintedanib [28], and its potency for inhibiting VEGFR-2 function may be greater.

As previously observed for sunitinib [74, 81], the combination of nintedanib with paclitaxel induced a significant anti-primary tumor effect (Fig. 1a). This effect translated in an improved median survival when LM2–4 advanced metastatic disease bearing mice were treated with the same two drugs (Fig. 3a). This stands in contrast to observations we previously reported when mice with LM2–4 advanced metastatic disease were treated with sunitinib combined with paclitaxel [74] (Fig. 3b). In a phase I clinical trial of HER2-negative breast cancer patients with early stage disease, the combination of nintedanib with conventional paclitaxel was evaluated, indicating a more tolerable toxicity profile [34] than previously observed for other antiangiogenic TKIs when they are combined with chemotherapy [13, 14, 17–19, 22–26, 31]. Indeed, adequate dose delivery with no necessary dose reductions, and no major side effects specific for antiangiogenic TKI drugs such as hypertension or hand–foot syndrome, were observed, albeit in a phase I trial [34]. Importantly, pathologic complete responses (pCRs) in 50% of patients were observed, including two out of two TNBC patients [34]. Despite the very small number of patients evaluated (*n* = 8) [34], this phase I study led to a phase II ‘window-of-opportunity’ neoadjuvant randomized trial involving monitoring hypoxia after which nintedanib was combined with paclitaxel [83].

In contrast to the modest toxicity reported in early stage HER2-negative breast cancer patients treated with nintedanib plus paclitaxel [34], in our preclinical study we observed signs of toxicity in the advanced metastatic treatment setting with LM2–4 cell line, occurring relatively soon after starting therapy with the drug combination. Thus, after 2 weeks of daily administration, the schedule was changed to a 5-days ON, 2-days OFF schedule. Such initial toxicity led to early treatment interruptions in some mice which may have influenced the survival data not reaching statistical significance despite a noticeable increase in median survival of mice treated with nintedanib plus paclitaxel compared to the control group (81 vs 60.5 days).

In view of the encouraging results of nintedanib combined with paclitaxel when treating mice with advanced (LM2–4) metastatic disease, we decided, during the course of these studies, to evaluate this drug combination using two mouse TNBC cell lines: EMT-6 and a derived drug-resistant variant, EMT-6/CDDP [77]. We observed that the modest tumor growth delay induced by nintedanib or paclitaxel monotherapy when treating primary EMT-6 or EMT-6/CDDP tumors was not improved when both drugs were administered together (Fig. 4a,b). This result differs from the antitumor effect induced by the same combination in LM2–4 human primary tumors grown in SCID mice (Fig. 1a). Such differences may be related, at least in part, to the greater aggressiveness and growth rate of mouse breast cancer cell lines, and possible differences in the molecular profile (ie. TNBC subtype) of the cell lines that would translate in differential response to the therapy [1, 6]. Unfortunately, identification of the molecular profile of TNBC mouse breast cancer cell lines used in preclinical studies is unknown. The MDA-MB-231 cell line has been previously classified as belonging to the mesenchymal-like subtype of TNBC [1].

It has been reported that taxanes do not affect, or can even promote, tumor infiltration of T lymphocytes in different cancer types [84, 85]. In breast cancer patients with advanced disease, treatment with taxanes systematically increased serum levels of various cytokines (eg. IFN-ɣ, IL-6 and GM-CSF) as well as the cytotoxic function of natural killer (NK) cells [86]. High levels of tumor-infiltrating lymphocytes have been correlated with response in breast cancer patients treated with neoadjuvant paclitaxel chemotherapy [87], particularly those with TNBC [56]. Moreover, some evidence suggests that taxanes may promote expression of PD-L1 by human breast cancer cells [88], which may then act to enhance the antitumor effect of a PD-L1 immune checkpoint inhibitor, as reported for metastatic TNBC patients [62].

We therefore decided to evaluate whether combining the nintedanib and paclitaxel doublet with a PD-L1 immune checkpoint antibody could improve overall anti-tumor activity in the syngeneic immunocompetent EMT-6 and EMT-6/CDDP mouse tumor models. We observed that EMT-6/CDDP cells express higher levels of PD-L1 in vitro than the parental EMT-6 cells [79]. In this study, we found that the PD-L1 antibody treatment induced a similar tumor growth delay, with respect to the control groups, when treating either EMT-6 or EMT-6/CDDP primary tumors (Fig. 4a,b). Based on these results, relative PD-L1 expression in vitro does not necessarily translate into differential sensitivity in vivo to PD-L1 therapy. Analysis in vivo of tumor samples prior to PD-L1 treatment is needed to determine whether similar response of those tumors to immunotherapy correlates with similar expression of PD-L1 in vivo.

We observed that nintedanib and paclitaxel improved the antitumor effect of PD-L1 antibody (and/or possibly vice versa) when administered in combination, compared to the control group, whether treating either primary EMT-6 (Fig. 4a) or EMT-6/CDDP tumors (Fig. 4b). Such a benefit may be related to an immunomodulatory effect of the TKI and the cytotoxic drug, based on the results obtained herein (Fig. 4c-f). Paclitaxel appeared to promote infiltration of CD8+ cells compared to the control group, although the result was not statistically significant (Fig. 4e). However, when adding nintedanib to paclitaxel (and to its combination with the PD-L1 antibody) such a trend disappeared (Fig. 4e). This suggests that, presumably, the improved antitumor effect of PD-L1 antibody when combined with nintedanib may be related to an immunomodulatory effect of the TKI ameliorating the VEGF-mediated intra-tumoral immunosuppressive microenvironment. Also, combining the PD-L1 antibody with paclitaxel significantly improved the antitumor effect of the chemotherapy on primary EMT-6 tumors (Fig. 4a). This preclinical result is in line with the improvement in PFS when the PD-L1 antibody atezolizumab was combined with nab-paclitaxel as a first-line treatment of metastatic TNBC patients, compared to nab-paclitaxel plus placebo, as recently announced on the basis of the phase III IMpassion130 clinical trial [63]. However, only nintedanib plus PD-L1 antibody treatment reached statistical significance in the EMT-6/CDDP model, compared to the control group. Thus, the antitumor effect of these combinations (ie. nintedanib or paclitaxel, plus PD-L1 antibody) seems to be influenced by the aggressiveness of tumor cells. On the other hand, the benefit of nintedanib combined with the PD-L1 antibody when treating EMT-6/CDDP primary tumors (Fig. 4b) did not translate into a prolonged median survival in the metastatic setting (Fig. 5). However, adding paclitaxel to nintedanib plus PD-L1 antibody (ie. using the triple drug combination) was the optimal treatment for improving the median survival of mice with metastatic TNBC. The reason why nintedanib or paclitaxel combined with the PD-L1 antibody showed efficacy when treating primary tumors, whereas only the triple combination caused prolonged median survival in the advanced metastatic setting using EMT-6/CDDP, is unknown. One possibility is that lung metastases (the main site of metastasis in our model) contain fewer tumor-infiltrating lymphocytes compared to primary tumors, as has been reported for human samples of metastatic lesions of TNBC at relapse compared to their matched primary tumors [89, 90]. Thus, in the advanced metastatic setting, contributions of the immunomodulatory roles of both nintedanib and paclitaxel to PD-L1 therapy seem necessary to increase median survival (Fig. 5). Results from this preclinical study are in line with the clinical benefit observed in a phase III clinical trial (IMpower150) in which the PD-L1 antibody atezolizumab was combined with bevacizumab and chemotherapy (the carboplatin and paclitaxel doublet) in metastatic NSCLC patients, compared to patients treated with bevacizumab plus chemotherapy (NCT02366143) [80]. Adding atezolizumab to the combination of bevacizumab, carboplatin and paclitaxel improved both PFS (8.3 vs 6.8 months) and OS (19.2 vs 14.7 months) [80].

Finally, we observed that PD-L1 antibody had a different safety profile when treating EMT-6/CDDP as primary tumors vs advanced metastatic disease. Toxicity events were more frequent in primary tumor-bearing mice, although less frequent than previously reported for the mouse breast cancer cell line 4 T1 [91]. Differential toxicity when treating EMT-6/CDDP primary tumors and advanced metastatic disease may be related to different tumor burdens. Mall et al. (2016) did not observe signs of toxicity when treating non-tumor bearing Balb/C mice with PD-L1 antibody (clone 10F.9G2, which was the same as used in this study); whereas 86% of 4 T1-bearing mice died after the third dose [91].

## Conclusions

Taken together, the results of this study suggest that combining nintedanib with conventional paclitaxel chemotherapy may be a potentially efficacious strategy to treat both primary and advanced metastatic TNBC, based on results using MDA-MB-231/LM2–4 model. Moreover, nintedanib plus paclitaxel therapy combination has also shown positive results in early stage HER2-negative breast cancer patients [34], as outlined in the Introduction. Furthermore, our results suggest that an antiangiogenic TKI, such as nintedanib, or MTD chemotherapy (using paclitaxel) may both improve the antitumor efficacy of PD-L1 antibody (and/or vice versa) when administered as a combination, in the primary tumor treatment setting, whereas, interestingly, the triple combination appears to be more effective when treating advanced metastatic TNBC compared to the standard MTD paclitaxel treatment. Finally, the results overall also once again reinforce the importance of analyzing preclinical therapy efficacy in different treatment settings, ie. not just conventionally treating primary tumors, but also metastatic disease, in addition to the use of multiple models/cell lines of the type of cancer under investigation.

## Additional files

Additional file 1: **Table S1.** Phase II and III Clinical trials evaluating Atezolizumab in combination with Bevacizumab (DOCX 15 kb)

Additional file 2: **Figure S1.** In vitro cell viability of MDA-MB-231, LM2–4, EMT-6 and EMT-6/CDDP cell lines when treated with increasing concentrations of paclitaxel. The IC_50_ values obtained for the different cell lines are: 5.41 ± 1.83 ng/mL for MDA-MB-231; 3.99 ± 0.78 ng/mL for LM2–4; 43.22 ± 6.08 ng/mL for EMT-6 and 4.73 ± 1.32 ng/mL for EMT-6/CDDP. The IC_50_ value for the EMT-6 cell line is significantly higher than for the other cell lines (*p* < 0.001). ANOVA followed by Tukey’s Multiple Comparison Test. (PNG 265 kb)

Additional file 3: **Figure S2**. Representative images of the primary tumors and lungs collected when mice injected with EMT-6/CDDP cells reached endpoint because of primary tumor volume. a) Primary tumors were stained with hematoxylin and eosin (H&E), showing a necrotic core, represented as a pink area resulting from eosin staining. b) Lungs stained with H&E; lung images show the presence of macrometastatic nodules across all the treatment groups at the time when mice reached endpoint because of large primary tumor volumes. Sections were visualized with a Leica MZFLIII microscope and digital camera (DFC300FX), magnification 8X. c) Only three mice did not have large metastatic nodules in the lungs visible with H&E staining. However, these mice had lung micrometastases visible with Ki67 staining (arrow head). Sections were visualized with a Leica DM LB2 microscope and digital camera (DFC300FX), magnification 100X. Bar represents 100 μm. Images were acquired using AxioVision 3.0 software. (PNG 7 kb)

## Abbreviations

ANOVA: Analysis of variance
DCs: dendritic cells
FBS: Fetal bovine serum
FGFRs: Fibroblast growth factor receptors
HER2: Human epidermal growth factor receptor 2
LAR: Luminal androgen receptor
MTD: Maximum-tolerated dose
Nint: Nintedanib
NK: natural killer
pCRs: Pathologic complete responses
PD-1: Programmed death 1
PD-L1: Programmed death-ligand 1
PTX: Paclitaxel
SCID: Severe combined immunodeficient
TKI: Tyrosine kinase inhibitor
TNBC: Triple negative breast cancer
VEGF: Vascular endothelial growth factor
YFP: Yellow fluorescent protein
