# Supplementary material for: Therapeutic impact of Nintedanib with paclitaxel and/or a PD-L1 antibody in preclinical models of orthotopic primary or metastatic triple negative breast cancer
Source: J Exp Clin Cancer Res. 2019 Jan 11;38:16. doi: 10.1186/s13046-018-0999-5 (PMC6330500; doi:10.1186/s13046-018-0999-5)
Supplement: Supplementary file 1 — Table S1. Phase II and III Clinical trials evaluating Atezolizumab in combination with Bevacizumab (DOCX 15 kb) [file 13046_2018_999_MOESM1_ESM.docx]

**Suppl. Table 1.** Phase II and III Clinical trials evaluating Atezolizumab in combination with Bevacizumab

| **Name of Study** | **Phase** | **Clinical trial ID** |
| --- | --- | --- |
| Treatment of Colorectal Liver Metastases With Immunotherapy and Bevacizumab [CLIMB] | II | NCT03698461 |
| FOLFOXIRI + Bev + Atezo vs FOLFOXIRI + Bev as First-line Treatment of Unresectable Metastatic Colorectal Cancer Patients [AtezoTRIBE] | II | NCT03721653 |
| Study With Atezolizumab Plus Bevacizumab in Patients With Chemotherapy Resistant, MSI-like, Colorectal Cancer | II | NCT02982694 |
| Capecitabine and Bevacizumab With or Without Atezolizumab in Treating Patients With Refractory Metastatic Colorectal Cancer | II | NCT02873195 |
| Atezolizumab With or Without Bevacizumab in Previously Untreated Metastatic/Unresectable Urothelial Cancer | II | NCT03272217 |
| Atezolizumab and Bevacizumab in Treating Patients With Recurrent, Persistent, or Metastatic Cervical Cancer | II | NCT02921269 |
| Triple-B Study; Carboplatin-cyclophosphamide Versus Paclitaxel With or Without Atezolizumab as First-line Treatment in Advanced Triple Negative Breast Cancer [Triple-B] | II | NCT01898117 |
| A Study of Atezolizumab (an Engineered Anti-Programmed Death-Ligand 1 [PD-L1] as Monotherapy or in Combination With Bevacizumab (Avastin®) Compared to Sunitinib (Sutent®) in Participants With Untreated Advanced Renal Cell Carcinoma [IMmotion150] | II | NCT01984242 |
| Phase II Study of Atezolizumab + Bevacizumab in Endometrial Cancer | II | NCT03526432 |
| Study of Bevacizumab in Combination With Atezolizumab in Patients With Untreated Melanoma Brain Metastases (BEAT-MBM) | II | NCT03175432 |
| Anti-programmed Cell Death-1 Ligand 1 (aPDL-1) Antibody Atezolizumab, Bevacizumab and Acetylsalicylic Acid in Recurrent Platinum Resistant Ovarian Cancer | II | NCT02659384 |
| Atezolizumab and Bevacizumab in Rare Solid Tumors | II | NCT03074513 |
| A Study of Atezolizumab Administered in Combination With Bevacizumab and/or With Chemotherapy in Participants With Locally Advanced or Metastatic Solid Tumors | II | NCT01633970 |
| A Study of the Safety and Efficacy of Atezolizumab Administered in Combination With Bevacizumab and/or Other Treatments in Participants With Solid Tumors | II | NCT02715531 |
| Atezolizumab With or Without Bevacizumab in Cisplatin-ineligible Patients with metastatic urothelial carcinoma | II | NCT03133390 |
| A study of Atezolizumab in Combination with Bevacizumab Compared With Sorafenib in Patients With Untreated Locally Advanced or Metastatic Hepatocellular Carcinoma [IMbrave150] | III | NCT03434379 |
| A Study of Atezolizumab in Combination With Bevacizumab Versus Sunitinib in Participants With Untreated Advanced Renal Cell Carcinoma (RCC) [IMmotion151] | III | NCT02420821 |
| A Study of Atezolizumab in Combination With Bevacizumab in Untreated Locally Advanced or Metastatic Clear Cell or Non-Clear Cell Renal Cell Carcinoma | III | NCT03693573 |
| Study of Atezolizumab + Bevacizumab in Patients With Advanced Non-Clear Cell Renal Cell Carcinoma | III | NCT02724878 |
| Combination Chemotherapy, Bevacizumab, and/or Atezolizumab in Treating Patients With Deficient DNA Mismatch Repair Metastatic Colorectal Cancer | III | NCT02997228 |
| Atezolizumab With Bevacizumab and Chemotherapy vs Bevacizumab and Chemotherapy in Early Relapse Ovarian Cancer | III | NCT03353831 |
| Pegylated Liposomal Doxorubicin Hydrochloride With Atezolizumab and/or Bevacizumab in Treating Patients with Recurrent Ovarian, Fallopian Tube, or Primary Peritoneal Cancer | III | NCT02839707 |
| Platinum Chemotherapy Plus Paclitaxel With Bevacizumab and Atezolizumab in Metastatic Carcinoma of the Cervix | III | NCT03556839 |
| A Study of Atezolizumab in Combination With Carboplatin Plus (+) Paclitaxel With or Without Bevacizumab Compared With Carboplatin + Paclitaxel + Bevacizumab in Participants With Stage IV Non-Squamous Non-Small Cell Lung Cancer (NSCLC) [IMpower150] | III | NCT02366143 |
