# Supplementary figures and images for: Therapeutic impact of Nintedanib with paclitaxel and/or a PD-L1 antibody in preclinical models of orthotopic primary or metastatic triple negative breast cancer
Source: J Exp Clin Cancer Res. 2019 Jan 11;38:16. doi: 10.1186/s13046-018-0999-5 (PMC6330500; doi:10.1186/s13046-018-0999-5)

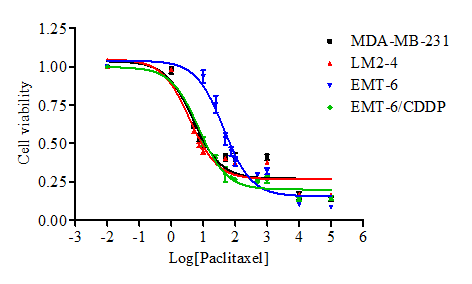

Supplement: Supplementary file 2 — Figure S1. In vitro cell viability of MDA-MB-231, LM2–4, EMT-6 and EMT-6/CDDP cell lines when treated with increasing concentrations of paclitaxel. The IC50 values obtained for the different cell lines are: 5.41 ± 1.83 ng/mL for MDA-MB-231; 3.99 ± 0.78 ng/mL for LM2–4; 43.22 ± 6.08 ng/mL for EMT-6 and 4.73 ± 1.32 ng/mL for EMT-6/CDDP. The IC50 value for the EMT-6 cell line is significantly higher than for the other cell lines (p < 0.001). ANOVA followed by Tukey’s Multiple Comparison Test. (PNG 265 kb) [file 13046_2018_999_MOESM2_ESM.png]

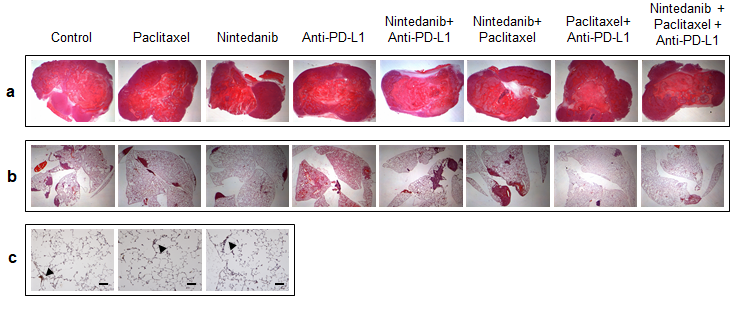

Supplement: Supplementary file 3 — Figure S2. Representative images of the primary tumors and lungs collected when mice injected with EMT-6/CDDP cells reached endpoint because of primary tumor volume. a) Primary tumors were stained with hematoxylin and eosin (H&E), showing a necrotic core, represented as a pink area resulting from eosin staining. b) Lungs stained with H&E; lung images show the presence of macrometastatic nodules across all the treatment groups at the time when mice reached endpoint because of large primary tumor volumes. Sections were visualized with a Leica MZFLIII microscope and digital camera (DFC300FX), magnification 8X. c) Only three mice did not have large metastatic nodules in the lungs visible with H&E staining. However, these mice had lung micrometastases visible with Ki67 staining (arrow head). Sections were visualized with a Leica DM LB2 microscope and digital camera (DFC300FX), magnification 100X. Bar represents 100 μm. Images were acquired using AxioVision 3.0 software. (PNG 7 kb) [file 13046_2018_999_MOESM3_ESM.png]
